# Role of Neuropilin-1 in Diabetic Nephropathy

**DOI:** 10.3390/jcm4061293

**Published:** 2015-06-17

**Authors:** Tzvetanka Bondeva, Gunter Wolf

**Affiliations:** Department of Internal Medicine III, University Hospital Jena, Jena, 07747, Germany; E-Mail: Tzvetanka.Bondeva@med.uni-jena.de

**Keywords:** NRP-1, diabetic nephropathy, AGEs, VEGF-A, Sema3A

## Abstract

Diabetic nephropathy (DN) often develops in patients suffering from type 1 or type 2 diabetes mellitus. DN is characterized by renal injury resulting in proteinuria. Neuropilin-1 (NRP-1) is a single-pass transmembrane receptor protein devoid of enzymatic activity. Its large extracellular tail is structured in several domains, thereby allowing the molecule to interact with multiple ligands linking NRP-1 to different pathways through its signaling co-receptors. NRP-1’s role in nervous system development, immunity, and more recently in cancer, has been extensively investigated. Although its relation to regulation of apoptosis and cytoskeleton organization of glomerular vascular endothelial cells was reported, its function in diabetes mellitus and the development of DN is less clear. Several lines of evidence demonstrate a reduced NRP-1 expression in glycated-BSA cultured differentiated podocytes as well as in glomeruli from *db/db* mice (a model of type 2 Diabetes) and in diabetic patients diagnosed with DN. *In vitro* studies of podocytes implicated NRP-1 in the regulation of podocytes’ adhesion to extracellular matrix proteins, cytoskeleton reorganization, and apoptosis via not completely understood mechanisms. However, the exact role of NRP-1 during the onset of DN is not yet understood. This review intends to shed more light on NRP-1 and to present a link between NRP-1 and its signaling complexes in the development of DN.

## 1. Introduction

Diabetic nephropathy (DN) is a well-known complication that occurs in diabetic patients with type 1 and type 2 diabetes mellitus in the course of disease [1,2,3]. DN is characterized by increased proteinuria and subsequently declining renal function [1,3]. Prolonged and uncontrolled hyperglycemia contributes to an accumulation of advanced glycation end-products [4,5,6], elevated angiotensin II (ANG II) levels, hypertension [7,8], chronic inflammation, and augmented generation of profibrotic cytokines such as transforming growth factor—beta1 (TGF-β1) [9]—as well as vascular endothelial growth factor-A (VEGF-A), all of which contribute to the development of DN [10]. Some of the most reported pathological changes in DN are a thickening of the glomerular basement membrane (GBM), glomerular hypertrophy, glomerulosclerosis, podocytes foot process effacement and increased podocytes loss, mesangial cells expansion, and tubulointerstitial fibrosis [1,2,11].

These pathophysiological changes result from altered gene and protein expressions of numerous targets or modulation of the physiological signaling cascades, thus leading to the onset and progression of DN. The neuropilins (NRPs), neuropilin-1 (NRP-1) and its homologue neuropilin-2 (NRP-2), are receptor molecules that bind various ligands via a large extracellular part that consists of several domains [12]. Although originally detected in neurons [13,14], neuropilins are also expressed in non-neuronal cells including renal cells [15,16,17,18]. Due to the absence of catalytic activity NRPs need co-receptors to transduce active signals into the cells, therefore they form complexes with a number of signaling co-receptors [12]. Albeit some of NRPs’ ligands and particularly those of NRP-1 are implicated in diabetes and DN, the role of NRP-1 is not yet completely elucidated. The aim of this review is to shed more light on the function of NRP-1 in culture podocytes and to present a link between NRP-1 and its signaling complexes in the development and progression of DN.

## 2. Neuropilin-1 Structure

**Figure 1:**
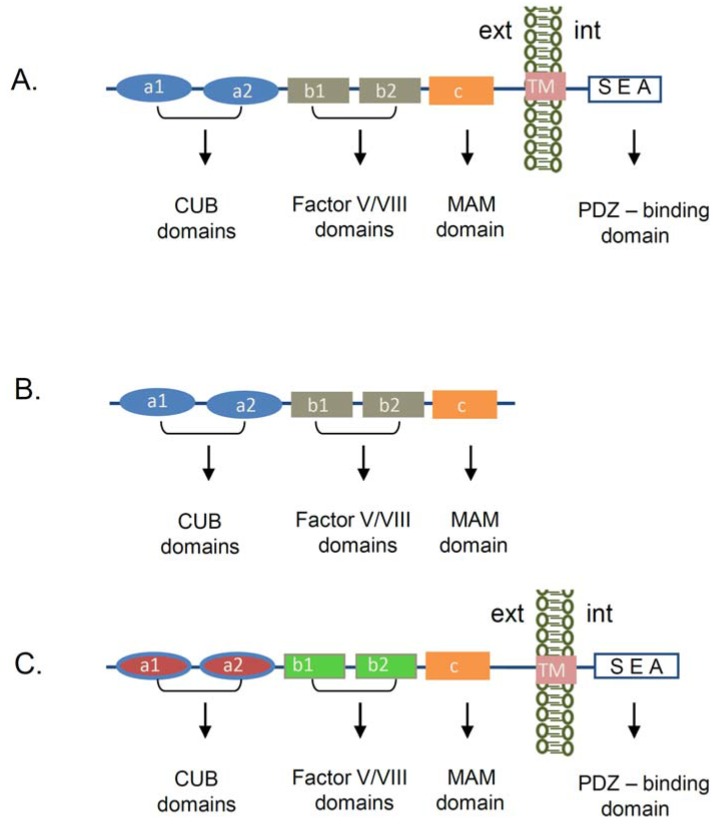
Schematic presentation of neuropilin’s structure. (**A**) Domain organization of the transmembrane Neuropilin-1 (NRP-1) receptor. The extracellular part of the NRP-1 receptor is organized in several domains. The a1/a2 domains (CUB domains) are required for the binding to semaphorin 3A (SEMA3A) ligand, while the b1/b2 domains (Factor V/VIII domains) are involved in the association with the Vascular Endothelial Growth Factor-A (VEGF-A). The c domain (MAM domain) plays an important role in the NRP-1 homophilic interaction and oligomerization. The transmembrane domain (TM) is important for dimerization and oligomerization of the protein. The last amino acids of the cytoplasmic part (SEA) confer a consensus sequence, which interacts with a PDZ-domain containing proteins. (**B**) Soluble NRP-1 receptor structure. The molecule consists of the same extracellular structure as the transmembrane NRP-1 receptor, but is missing the TM and the cytoplasmic part of the NRP-1 receptor. (**C**) NRP-2 transmembrane protein—domain organization. Both NRP-1 and NRP-2 share 44% amino acid homology. NRP-2 consists of the same domains as NRP-1 but binds different signaling ligands and co-receptors. (CUB—C1s/C1r, Uegf (urchin embryonic growth factor), and the Bone morphogenic protein 1 (BMP1), MAM—mephrin, A5 antigen, receptor tyrosine phosphatase—μ, PDZ—PDS-95/Dlg/ZO-1 domain, TM—transmembrane, ext—extracellular environment, int—intracellular environment).

Neuropilin-1 (NRP-1) was first discovered as an antigen that binds to the A5-antibody, raised against neuronal cell surface proteins in the nervous system [19]. It was originally reported that NRP-1 functions as an adhesion receptor in the nervous system [13]. Subsequently, studies revealed that NRP-1 is implicated into the axon guidance through its association with semaphorin III (Sema III) family of proteins [20,21,22]. In addition to the nervous system the expression of NRP-1 was also reported in the heart [23], endothelial cells [24], tumor cells [25], stromal cells [26], and T cells [27,28]. We and others also detected NRP-1 mRNA and protein expression in renal cells [15,16,17,18]. NRP-1 is a transmembrane protein, consisting of a large extracellular part and a short intracellular tail. While its domain structure shares about 44% amino acid homology and structural similarity with the neuropilin-2 (NRP-2) protein, the molecules differ in regard to their function and ligand binding [14] (Figure 1). The NRP-1 extracellular region is organized in five domains: two CUB domains, two Factor V/VIII domains, and a MAM domain, followed by a transmembrane domain and an intracellular tail (Figure 1A). The CUB domains (a1/a2) share homology with complement binding factors C1s/C1r [29], Uegf (urchin embryonic growth factor) [30], and the bone morphogenic protein 1 (BMP1) [31]. In the complement system the CUB domains are known to induce protein-protein interactions, which are mainly regulated by the formation of the immunoglobulin-like structures. Several CUB-domain containing proteins were implicated in the regulation of the cellular adhesion and motility [32,33]. Structural studies have shown that a1/a2 domains of NRP-1 participate in the binding to Semaphorin ligand(s) [34], while the two Factor V/VIII (b1/b2) domains promote the association with another NRP-1 ligand VEGF-A_164_ in mouse or VEGF-A_165_ in humans [35]. The NRP-1 extracellular domain crystal structure revealed that the b1 domain is necessary for the VEGF-A_164_ binding, whereas the b2 domain is mainly involved in the stabilization and coordination of the binding between NRP-1 and its ligands Sema3A or VEGF-A_164_ [34]. The so named MAM (**c**) domain, displays homologies with the extracellular regions of mephrin [36], A5 antigen, and the receptor tyrosine phosphatase—µ [37]. This part of the protein is hypothesized to play an important role in the NRP-1 homodimerization, due to its capacity to induce homophilic interactions [38]. Structural studies of the transmembrane part of the NRP-1 depicted a putative GxxxG motif, which is thought to participate in receptor dimerization or oligomerization [39]. The intracellular SEA amino acid sequence in NRP-1,2 is a consensus region shown do promote association with the PSD-95/Dlg/ZO-1 (PDZ) domain containing proteins as the neuropilin interacting protein-1 (NIP1) termed also synectin or RGS-GAIP-interacting protein (GIPC) [40].

In addition, NRP-1 is also presented in a soluble form, missing the cytoplasmic tail and the transmembrane region of the molecule (Figure 1B). Its role is not well characterized, but it is suggested that it can function as a decoy receptor for NRP-1 ligands [41].

## 3. Neuropilin-1 Ligands and Signaling Co-Receptors

**Figure 2:**
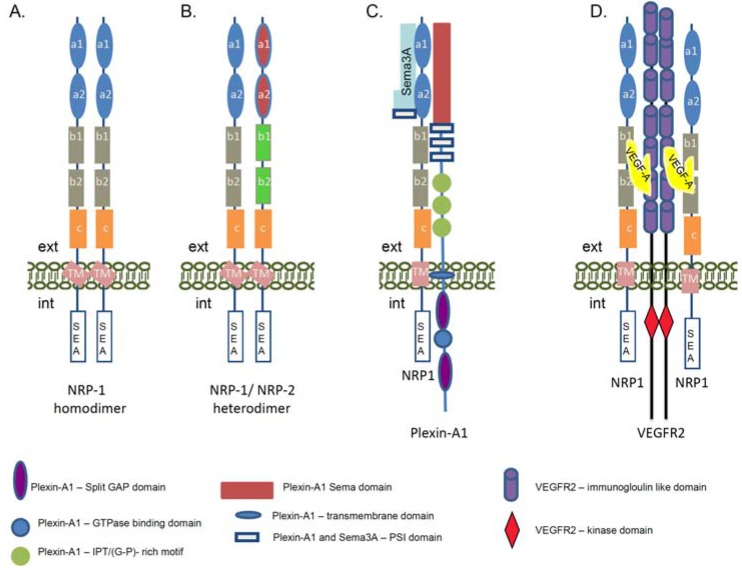
Schematic presentation of the neuropilin-1 signaling complexes. The cartoon represents the complex formation between NRP-1, its ligands, and signaling co-receptors. (**A**) Formation of the homodimers between the NRP-1 molecules. The homodimerization of NRP-1 receptors can occur via the transmembrane domain of the proteins. The domains of the NRP-1 receptor are described in Figure 1A; (**B**) Heterodimerization between NRP-1 and NRP-2 receptors. The domains of the NRP-2 receptor are described in Figure 1C; (**C**) Structure of the functional Sema3A/NRP-1/Plexin-A1 receptor. Via this signaling complex NRP-1 is involved in the regulation of axon guidance signaling in neuronal cells and promotes repulsion signals. The complex is functionally activated upon association of NRP-1 with semaphorin 3A (Sema3A). NRP-1 a1 and a2 domains are involved in the association with Sema3A. Plexin-A1 is a single pass transmembrane protein. In the extracellular part it contains a Sema domain, a PSI motif, as Sema3A and IPT (G-P)-rich motifs. The intracellular part contains a split GAP domain separated by a GTP-ase binding domain. (**D**) Schematic presentation of the NRP-1 and Vascular Endothelial Growth Factor Receptor 2 (VEGFR2) holoreceptor complex. The interaction between NRP-1 receptor and VEGFR2 co-receptor is regulated via VEGF-A association, but the binding of NRP-1 to VEGFR2 enhances the signaling activity of the VEGFR2 tyrosine kinase receptor. The VEGF-A_164/165_ binds to the b1/b2 domains of the NRP-1 and this association enhances the VEGFR2 tyrosine autophosphorylation. VEGF-A binds to both receptors and is a crosslink between NRP-1 and VEGFR2. Via this signaling complex NRP-1 is involved the regulation of angiogenesis signaling as well as adhesion and migration of endothelial cells. (ext—extracellular environment, int—intracellular environment, GAP—GTP-ase activating protein, G-P (glycin-proline), IPT—immunoglobulin-plexin-transcription, PSI—plexin, semaphorin, integrin, VEGF—Vascular Endothelial Growth Factor, Sema3A—semaphorin 3A).

The well-structured extracellular part of the NRP-1 receptor suggests its involvement in numerous extracellular interactions and signaling pathways. NRP-1 has no catalytic activity, therefore to transduce signals into the cells it must associate with multiple ligands and signaling co-receptors (Figure 2A–D). In neurons, NRP-1 is essential for distribution of the down-stream signals initiated from class III semaphorin (Sema3) family of axon guidance molecules [20,21,41,42]. NRP-1 specifically binds to Sema3A [43], while its homolog NRP-2 interacts with Sema3F [44]. The formation of heterodimers between NRP-1 and NRP-2 receptors can also link NRP-1 to Sema3C signaling [44] (Figure 2B). Although NRP-1 binds Sema3A with high affinity, this interaction cannot mediate a functional signaling cascade. The discovery of the Plexins as a binding partner of NRPs revealed that the function of NRP-1 is to bridge Sema3A to the Plexin-A1 co-receptor in order to generate a physiologically active holoreceptor complex regulating the axon guidance [45] (Figure 2C). Plexin-A1 alone does not associate with the Sema3A ligand, but the NRP-1/Plexin-A1 complex has a higher affinity and specificity for Sema3A compared with NRP-1 alone [45,46]. It is shown that Plexin-A1 activation is directly involved in the guidance of axonal growth and induced a Plexin-A1-regulated cytoskeleton collapse, causing an axon repulsion of the growth cone [41,46]. On the other hand, in the presence of a high cGMP level the Sema3A can converse the down-stream signals from repulsion to attraction in neuronal growth cone [47].

In addition to Sema3A signaling, NRP-1 plays an important role in endothelial cells via its binding to Vascular Endothelial Growth Factor A (VEGF-A), one of the most potent pro-angiogenic cytokines of the VEGF family [35,48,49]. In similarity with the nervous system in endothelial cells, NRP-1 needs the VEGFR1 or VEGFR2 signaling co-receptors to fulfill its function [24,35] (Figure 2D). VEGF receptors belong to the family of receptor tyrosine kinases and are implicated in normal embryonic development and pathological angiogenesis [50]. They can trap the VEGF ligands but the binding of VEGF-A_165_/VEGF-A_164_ to NRP-1 enhanced the VEGFR2 activity and tyrosine phosphorylation and elevated endothelial cells migration [51,52]. Currently, it is known that a strong coordination between VEGF-A, VEGFR2, and NRP-1 molecules is essential for angiogenesis [53,54], as a deletion of NRP-1 in mice failed to activate the pro-angiogenic signaling path due to defective vasculature formation [55]. Studies in human umbilical vein endothelial cells revealed that NRP-1 association with VEGFR2 is VEGF_165_-dependent as confirmed by co-immunoprecipitation assay [51]. Moreover, the complex between NRP-1 and the VEGFR2 can be assembled in *cis* (both receptors are expressed in the same cell) as well as in *trans* (the complex is formed between receptors present on different cells) [51]. Thus, the NRP-1 receptor could function as an extracellular scaffold molecule generating cell–cell crosstalk communications and cross-signaling.

During the last decade the involvement of NRPs in tumor biology [56] and immunology [57] was investigated. It has been reported that NRP-1 is implicated in the signaling events downstream of transforming growth factor beta (TGF-β1) [27,58,59], or platelet-derived growth factor (PDGF) [58,60] and the number of NRP-1 signaling receptor complexes is constantly growing. Many of the signaling cascades related to NRP-1 function in different cell types need to be further studied as the mechanisms are not well understood. NRP-1 was also very recently reported as a molecule able to transport other large molecules into the cells and may play a cargo role [61].

NRP-1 participates in molecular interactions through its intracellular SEA consensus domain that recruits to NRP-1 PDZ-domain containing proteins. The best characterized is synectin [40]. Synectin is involved in the arteriogenesis [62] and facilitation of the trafficking of endocytosed membrane receptors VEGFR2 and NRP-1 [49,63]. A recent study demonstrated that NRP-1 in endothelial cells regulates the focal adhesion turnover through its association with p130Cas [64].

## 4. NRP-1 Expression in Diabetes and Diabetic Nephropathy

**Figure 3:**
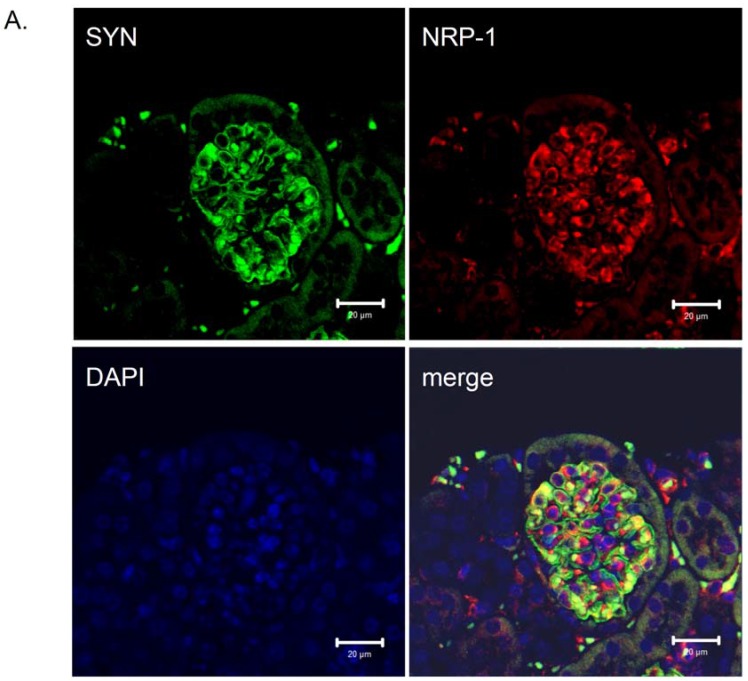

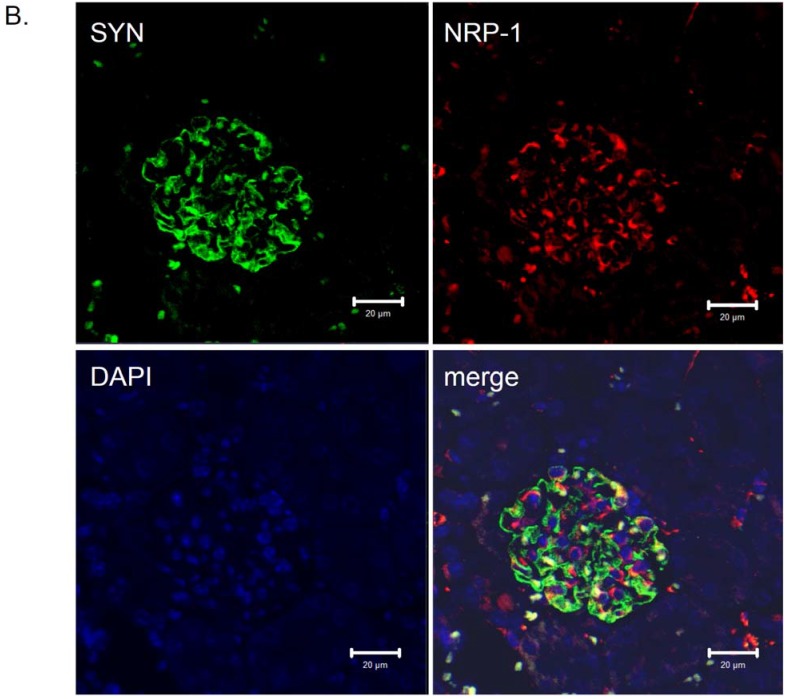
Distribution of neuropilin-1 protein in kidney glomeruli of diabetic *db/db* mice and non-diabetic *db/m* littermates. Double immunological detection of neuropilin-1 (NRP-1) and synaptopodin (SYN), a podocyte specific marker protein. Nuclei were counterstained with DAPI. Images were analyzed using LSM 510 META and ZEN 2009 software (Zeiss, Germany). The staining was performed on 2 µm paraffin kidney sections originated from diabetic *db/db* mice and non-diabetic *db/m* littermates. The co-localization of the NRP-1 and synaptopodin proteins is presented as a merge image. Bars correspond to 20 µm. Magnification 400x. (**A**) Protein expression in glomeruli of non-diabetic *db/m* mice. (**B**) Protein expression in glomeruli of diabetic *db/db* mice. The expression of NRP-1 is reduced in *db/db* mice (**B**) compared with non-diabetic *db/m* littermates (**A**). The NRP-1 stain is co-localized with synaptopodin, as seen on the merged images.

The expression of neuropilin-1 mRNA and protein in renal cells was previously described [15,16,17,18,65]. Villegas and Tufro reported that NRP-1 expression did not change in cultured undifferentiated and differentiated podocytes [18,65]. We detected that NRP-1,2 are highly expressed in differentiated podocytes [17,66]. Moreover, the immunohistochemistry staining for NRP-1 in glomeruli localized this protein to podocytes [17,67]. The presence of NRP-1 in differentiated podocytes was also confirmed *in vitro* and *in vivo* by Robert *et al.* [15]. Due to prolonged hyperglycemia in diabetic patients, accumulation of advanced-glycation end-products (AGEs) is highly increased [5]. Glomerular podocytes are a target of AGEs in diabetes through an elevated expression of their receptor RAGE [68] and AGEs/RAGE axis activation. Recently, we identified *Nrp-1* as a downregulated gene in cultured differentiated podocytes due to glycated-BSA exposure [17,66]. Moreover, we detected a reduced NRP-1 protein expression in the glomeruli of diabetic *db/db* mice, an animal model to study DN [17,67] (Figure 3) (original figure) and in kidney biopsies from patients with DN [17]. A recent study demonstrated that treatment with epoetin-β or continuous erythropoietin receptor activator (CERA) of diabetic *db/db* mice correlated with a reduced albuminuria and increased expression of NRP-1 in treated animals compared with the non-treated [67]. These data support the observation that reduced NRP-1 expression is a characteristic of DN, and reversing/preventing the injury of podocytes is associated with a regain of NRP-1 expression [67]. Using a reporter assay analysis, we found that the regulation of NRP-1 in cultured differentiated podocytes was under the control of the Sp-1 transcription factor as mutations of the Sp-1 sites on the NRP-1 promoter completely abolished its activity [66]. Our data also confirm the findings of Rossignol *et al.* [69], showing a similar regulatory mechanism of the NRP-1 promoter in HeLA cells [69]. In agreement with our previous finding, glycated-BSA inhibited NRP-1 promoter activity, thus reducing the *Nrp-1* gene expression in differentiated podocytes [17,66] through reduced binding of the Sp1-transcription factor to the NRP-1 promoter [66]. Furthermore, a TGF-β1 dependent downregulation of NRP-1 but not NRP-2 expression was also reported in human proteinuric nephropathies and cytokine-stimulated proximal tubular cells in a TGF-β1-dependent manner [70].

## 5. NRP-1 Is Implicated in Podocyte Adhesion and/or Migration

In DN proteinuria develops as a result of effacement of the podocyte foot processes or podocytes loss due to apoptotic events and a nude GBM is generated [71,72]. Podocytes are often found in the urine of patients with DN [71,73], but it is difficult to characterize them as apoptotic podocytes as the cells are viable and could be further cultured *in vitro* [74]. Thus, it is possible that the podocyte loss is a result of the weakening of their adhesion ability to GBM. Interestingly, previously we found in *in vitro* studies (using cultured differentiated podocytes) that treatment of the cells with glycated BSA (AGE-BSA), or reduction of the NRP-1 expression by NRP-1 siRNA, both were associated with a reduced adhesion ability of the cells to different extracellular matrixes (ECM), e.g., collagen IV, fibronectin, laminin, all of which are characteristic for the GBM [75]. Furthermore, a forced overexpression of NRP-1 reversed the adhesion capacity of podocytes to the ECM even at the presence of glycated BSA [75]. We found also that this process was accompanied by a reduced activation of the small GTPases Rac1 and Cdc42 and was manifested in cytoskeleton dysfunction, which was NRP-1 dependent [75]. Intriguingly, a recent study in HEK 293 cells unveiled a new function of collagen IV, showing that it specifically associates with the extracellular amino-terminal region of Gpr126 adhesion receptor, which is a G-protein coupled receptor, containing the CUB (complement, Uegf, Bmp1) domain [33]. This finding raises the possibility that the reduced adhesion we observed in cultured podocytes could be explained with decreased direct association between collagen IV and NRP-1 CUB domains (see Figure 1) as a result of reduced NRP-1 protein expression [75]. Furthermore, the study of Paavola *et al.* [33] suggests a new function of collagen IV in the basement membranes as a signaling component. At present it is unclear how NRP-1 regulates glomerular podocytes adhesion and migration processes and which signaling receptors are involved. Indeed, in endothelial cells it was demonstrated that NRP-1 has as well a VEGFR2 independent function in the regulation of endothelial cell adhesion and spreading to fibronectin and fibrilogenesis via its cytoplasmic PDZ-containing protein binding domain and association with the GIPC1 protein [76].

## 6. NRP-1 Ligand Sema3A and Its Function in DN

Semaphorins are a large class of secreted axon chemorepelents that are involved in axon guidance, cell adhesion, migration, invasion, and proliferation signaling paths via interaction with their receptors NRPs and plexins [77]. The holoreceptor Neuropilin-1/Plexin-A1 mediates cellular signals specifically via its ligand semaphorin 3A (Sema3A) [45]. In the kidney, Sema3A is expressed in the developing nephrons, differentiated podocytes, and collecting tubules [18]. It was reported that Sema3A is a negative regulator of the ureteric bud branching morphogenesis [78]. Studies in *Sema3a*^−/−^ transgenic mice revealed that it is essential for glomerular development, because the absence of *Sema3a* was associated with defects in renal vascular patterning, increased number of endothelial cells within glomerular capillaries, effaced podocytes foot processes, and development of albuminuria [79]. On the other hand, podocyte-specific *Sema3a* overexpression in mice resulted in renal dysfunctions, revealing severe podocyte and endothelial cell damage and/or apoptosis during organogenesis as represented by glomerular hypoplasia, impaired podocyte foot processes development, completely missing podocytes slit diaphragms, congenital proteinuria, decreased Nephrin, WT1 (Wilms tumor 1), and VEGFR2 expression [79]. Interestingly, the alteration of *Sema3a* expression in podocytes was not associated with the modulation of the NRP-1 receptor expression [79]. These data suggest an important function of Sema3A in vascular morphogenesis and podocytes endothelial crosstalk and the formation of the glomerular filtration barrier. Taken together, the manipulation of the *Sema3a* expression up- or downregulation impaired the glomerular function. Nevertheless, as NRP-1 is the main Sema3A link to its signaling unit, the Plexin-A1 co-receptor, at present the exact signaling mechanism coordinating all these processes in unclear. Interestingly, in *in vitro* studies using differentiated podocytes we found that suppression of NRP-1 expression using siRNA transfection induced podocytes apoptosis [17] and deletion of *Sema3a* in podocytes *in vivo* also affected podocytes survival [79]. It was also shown that an overexpression of *Sema3a* in podocytes *in vivo* was associated with glomerular disease, via deregulation of the nephrin/Plexin-A1 interaction thus linking Sema3A and Plexin-A1 to SD complexes [80].

A very recent study further elucidated the role of Sema3A in DN as the authors demonstrated that Sema3A promotes diabetic nephropathy [81,82]. They reported that mice caring a podocyte-specific gain-of-function *Sema3a* developed massive proteinuria and experienced declining renal function and an exacerbation of the ECM protein as laminin and collagen IV accumulation [82]. In-depth studies showed that all these processes resulted from podocytes foot process effacement and F-actin collapse and were regulated via nephrin, αVβ3 integrins, and MICAL1 association with the Plexin-A1 signaling receptor [82]. This is a very interesting observation but the direct association between the Plexin-A1 signaling co-receptor and Sema3A ligand is not demonstrated, whereas NRP-1, which is the bridge between the Sema3A and Plexin-A1 signaling receptor, interacts directly with the Plexin-1 and does not need Sema3A for this binding [45,46]. In other words, the complex between NRP-1 and Plexin-A1 already exists in the cells that are expressing both molecules and is activated via association of the NRP-1 with a Sema3A ligand. Nevertheless, collectively all these reports revealed an essential role for Sema3A/NRP-1(?)/Plexin-A1 signaling in development of DN and modulation of the complex is inducing pathological changes in the glomerular podocytes.

## 7. NRP-1 Ligand VEGF-A and Its Function in DN

VEGF-A is highly expressed and secreted from glomerular podocytes. It is assumed that it crosses the glomerular basement membrane (GBM) and transduces signals via its own receptor VEGF receptor 2 (VEGFR2) on endothelial cells [83]. The VEGF-A/VEGFR2 complex formation is implicated in the formation and maintenance of the glomerular filtration barrier, as genetic manipulation of the *Vegf-a* expression in glomeruli is associated with glomerular disease in mice [84] and a podocyte-specific deletion of *Vegf-a* expression is characterized by impaired recruitment of the endothelial cells into glomeruli, failure of the glomerular filtration barrier formation, and congenital nephrotic syndrome [84]. However, lately it was shown that in addition to the paracrine VEGF-A signaling in glomerulus, there is evidence for autocrine VEGF-A effects, which support podocytes’ survival [85,86,87] and also link nephrin to VEGF-A signaling in podocytes [85]. VEGFR2 expression in podocytes is somewhat contradictory, but at least in cultured differentiated podocytes Foster *et al.* convincingly demonstrated VEGFR2-dependent effects [85]. Interestingly, we also found that the expression of the VEGFR2 in cultured differentiated podocytes is very weak and detectable by real-time PCR but not by western blot analysis [17]. Furthermore, the treatment of cultured differentiated podocytes with glycated-BSA induced the expression of VEGFR2, while it inhibited NRP-1 expression [17].

## 8. Linking Neuropilin-1 to the Slit Diaphragm Proteins via CD2AP/NRP-1 Complex?

Localization of the NRP-1 protein to the slit diaphragm has not yet been demonstrated but based on the known NRP-1 interaction partners and its molecular structure we would like to present some evidence for a possible participation of the NRP-1 in this complex. Slit diaphragm is a specialized structure formed between the interdigitating individual foot processes of the glomerular podocytes to maintain a constant distance between each of the processes at the opening of the urinary space [88]. Rodewald and Karnovsky, some 30 years ago, based on their electron microscopic analysis suggested that slit diaphragm has an isoporous zipper-like structure [89]. Several proteins were reported to play an important role in the formation of this structure: nephrin was suggested to be the main structural component of the slit diaphragm [90,91,92], along with Zona occludence-1 (ZO-1) [93], P-cadherin [94], β-catenin [95], FAT1 [96,97], Neph1 [98] and CD2AP [99]. Due to its large extracellular tail, NRP-1 can be a docking place for a number of different ligands but it can also participate in many homophilic and heterophilic complexes. The main structural component of the slit diaphragm, the nephrin molecule, is suggested to be bridged to the podocytes’ cytoskeleton through its interaction with the CD2-associated protein (CD2AP) [99]. In the kidney CD2AP is mainly detected in glomerular podocytes [100]. The CD2AP knockout mice die at six to eight months of age due to development of nephrotic syndrome [101]. Detailed analyses of the CD2AP function in podocytes unveiled that it is localized in close proximity with the slit diaphragm and is associated with nephrin, as demonstrated by co-immunoprecipitation studies from a differentiated podocyte cell line [99]. In endothelial cells CD2AP is found to bind to the α3/βV integrins and form a large complex also involving NRP-1, thus providing evidences that both CD2AP and NRP-1 are linked to the extracellular matrix. Furthermore, NRP-1 molecules have the ability to form dimers from adjacent cells and it could be expected as well that due to their large extracellular part they can probably form homo- or heterodimers with molecules from the “adjacent” podocytes’ foot processes, *i.e.*, they can “bridge” the interdigitated foot processes, as the *cis*- and *trans*-dimers for NRP-1/VEGFR2 are reported, as discussed above.

## 9. Conclusions

DN is associated with severe renal abnormalities inducing proteinuria resulting ultimately in glomerulosclerosis, tubulointerstitial fibrosis, and extracellular matrix. Multiple factors contribute to the development of DN. High glucose levels can modify proteins, lipids, and amino acids to generate advanced glycation end-products (AGE) which are one of the key factors involved in the onset of DN. Furthermore, elevated ANG II hypertension, oxidative stress, and increased cytokine production alter the physiological signaling, induce pathological processes, and impair the function of the renal cells. During the last decades of research a lot of knowledge was accumulated in regard to the molecular mechanisms involved in the onset and development of DN. Since progressive proteinuria is one of the main characteristics of DN, studies on glomerular podocytes received a large amount of attention because podocytes are the key component maintaining the glomerular filtration barrier via formation of the slit diaphragms and GBM in concert with glomerular endothelial cells. Due to its multidomain structure, NRP-1 is capable of binding many ligands and transducing cellular signals via its co-receptors into the cells. Its direct function in DN is not as well understood as is its function as an adhesion receptor, regulating the neuron repulsion via an association to Sema3A and Pexin-A1 signaling co-receptor in the nervous system or as an important regulator of angiogenesis in endothelial cells through interaction with VEGF-A_164/165_ and VEGFR2 co-receptor complex. In cultured differentiated podocytes NRP-1 expression is suppressed during the exposure to AGEs, a key factor known to induce pathophysiological changes of DN. Furthermore, NRP-1 reduction was at least in part associated with a declined migration and adhesion ability of the podocytes to GBM extracellular matrix components such as collagen IV, fibronectin, and laminin, impaired cytoskeleton reorganization, podocytes apoptosis, and a lower GTP-binding activity of the small Rho-GTPases Rac1 and Cdc42. NRP-1 expression was also decreased in podocytes from diabetic *db/db* mice as well as in diabetic patients diagnosed with DN. Interestingly, epoetin-β and CERA treatment of *db/db* mice, which reduced proteinuria, also increased the NRP-1 protein expression in podocytes. Thus, the generation of podocyte-specific deletion and overexpression of NRP-1 using animal models will shed more light onto the role of NRP-1 in diabetic disease and particularly in the development of DN.
